# Brassinosteroids Positively Regulate Plant Immunity *via* BRI1-EMS-SUPPRESSOR 1-Mediated *GLUCAN SYNTHASE-LIKE 8* Transcription

**DOI:** 10.3389/fpls.2022.854899

**Published:** 2022-03-24

**Authors:** Jiawei Xiong, Xiaoping Wan, Maolin Ran, Xiumei Xu, Lezhang Chen, Feng Yang

**Affiliations:** ^1^Key Laboratory of Southwest China Wildlife Resources Conservation (Ministry of Education), China West Normal University, Nanchong, China; ^2^Rice and Sorghum Research Institute, Sichuan Academy of Agricultural Sciences, Deyang, China; ^3^Innovative Institute of Chinese Medicine and Pharmacy, Chengdu University of Traditional Chinese Medicine, Chengdu, China; ^4^Vegetable Germplasm Innovation and Variety Improvement Key Laboratory of Sichuan Province, Chengdu, China; ^5^State Key Laboratory of Crop Stress Adaptation and Improvement, Key Laboratory of Plant Stress Biology, School of Life Sciences, Henan University, Kaifeng, China; ^6^Sichuan Huitai Agriculture Technology Co. Ltd., Chengdu, China

**Keywords:** brassinosteroids, *Pst* DC3000, *Arabidopsis thaliana*, plant immunity, callose deposition

## Abstract

Plant hormone brassinosteroids (BRs) play key roles in plant adaptation to biotic stresses, including various pathogen infections. As a core factor in BR signaling, the transcription factor BRI1-EMS-SUPPRESSOR 1 (BES1) activates BR responses *via* regulating the expression of target genes. However, the molecular mechanism of BRs in regulating plant immunity is unclear, and the key components are not identified. In this study, we found that BR biosynthesis and signaling transduction are essential for plant resistance to pathogen infection, and BR biosynthesis or BR signaling-deficient mutants displayed susceptibility to *Pseudomonas syringae* pv. *tomato* DC3000 (*Pst* DC3000) infection [including more serious symptoms and more photosystem II (PSII) photochemistry damage]. We identified a callose synthase gene *GLUCAN SYNTHASE-LIKE 8* (*GSL8*) as a direct target of BES1, and its expression was induced by BRs/BES1. Meanwhile, BRs induced callose accumulation after *Pst* DC3000 infection. Moreover, BES1 gain-of-function mutant *bes1-D* showed promoted *Pst* DC3000 resistance. *GSL8* T-DNA insertion mutant *gsl8-1* was susceptible to DC3000, while brassinolide (BL) treatment partially rescued *gsl8-1* susceptible phenotypes. Our study suggests that BR-induced pathogen resistance partly depends on the BR-induced BES1-GSL8 cascade to mediate callose accumulation.

## Introduction

One-upmanship competition between plants and pathogens has been going on for millions of years. Sessile plants have evolved a dynamic defense regulatory network to survive from pathogen attacks. The plant rapidly activates defense response after perceiving pathogen attack (Robert-Seilaniantz et al., 2011). There are two key interconnected branches in plant immunity (Jones and Dangl, 2006); at first, pathogen-associated molecular patterns (PAMPs) or host-derived damage-associated molecular patterns are perceived by pattern recognition receptors (PRRs) which can lead to pattern-triggered immunity (PTI) (Zipfel, 2014), which can resist most of the attacks. However, pathogens have evolved an ability that delivers effectors to suppress PTI (Chisholm et al., 2006; Jones and Dangl, 2006; Goehre and Robatzek, 2008). In response, plants have acquired resistance (*R*) genes that can recognize these attacker-specific effectors, resulting in effector-triggered immunity (ETI) (Chisholm et al., 2006; Jones and Dangl, 2006). Phytohormones play essential roles during pathogen infections. Salicylic acid (SA), jasmonic acid (JA), ethylene (ET), cytokinin (CK), abscisic acid (ABA), gibberellic acid (GA), auxins, and brassinosteroids (BRs) are known to primarily regulate the basal defense responses (Nemhauser et al., 2006), and the hormone signaling works synergistically or antagonistically in plant–microbe interactions (Verhage et al., 2010).

Brassinosteroids, a kind of plant steroid hormones, play essential roles during plant growth and development such as hypocotyl and petiole elongation, leaf senescence, vascular development, and stress response (Clouse, 1996; Yang et al., 2011; Nolan et al., 2017). BR signaling pathway is well understood. BRs are perceived by receptor kinase BR INSENSITIVE 1 (BRI1) and co-receptor BRI1-ASSOCIATED KINASE 1 (BAK1) (Li and Chory, 1997; Li et al., 2002; Nam and Li, 2002; Hothorn et al., 2011; She et al., 2011; Gou et al., 2012). Then, BR signaling is transmitted from the plasma membrane to nuclear by other components, including BRI1 SUPPRESSOR 1 (BSU1), BRASSINOSTEROID-INSENSITIVE 2 (BIN2), BRASSINAZOLE-RESISTANT 1/BRI1-EMS-SUPPRESSOR 1 (BES1/BZR1), MYB-LIKE 2 (MYBL2), HOMEODOMAIN-LEUCINE ZIPPER PROTEIN 1 (HAT1), UPBEAT 1 (UPB1), and GOLDEN2-LIKE 1 (GLK1) to regulate up to 4,000–5,000 gene expressions (Yin et al., 2002; He et al., 2005; Sun et al., 2010; Kim et al., 2011; Yu et al., 2011; Ye et al., 2012; Zhang et al., 2014, 2021; Li et al., 2020). Several studies have been indicated that BRs participate in the pathogen defense process (Heese et al., 2007; Albrecht et al., 2012; Belkhadir et al., 2012). A previous study demonstrated that the MITOGEN-ACTIVATED PROTEIN KINASE 6 (MEK6) phosphorylates BES1 to enhance plant immunity (Kang et al., 2015). BAK1 plays as a partner of FLAGELLIN SENSING 2 (FLS2) or PEPTIDE 1 RECEPTORS (PEPRs) to function in PTI (Chinchilla et al., 2007; Sun et al., 2013). RECEPTOR-LIKE CYTOPLASMIC KINASES (RLCK) group VII members, BR-SIGNALING KINASE 1 (BSK1) and BSK5, play essential roles in PTI (Shi et al., 2013; Majhi et al., 2019; Zhao et al., 2019; Wang et al., 2020). Recent studies have also found important roles of BRs in the plant antivirus process (Deng et al., 2015, 2016; Zhang et al., 2015). However, the molecular mechanism of BRs in regulating plant immunity is unclear, and the key component is not identified.

Callose is a β-(1,3)-D-glucan polymer, and callose deposition is a typical PTI response (Ellinger and Voigt, 2014; Voigt, 2016). Callose deposits on the site of pathogen infection to restrict the ingression of pathogen-secreted cell wall-degrading enzymes (Stone, 2009). Callose is involved in various plant developmental processes and stress responses, and its biosynthesis is regulated by the family of *GLUCAN SYNTHASE-LIKE* (*GSL*) genes (Ellinger and Voigt, 2014). After pathogens attack, callose is deposited between the plasma membrane and the cell wall (Nishimura et al., 2003). A recent study indicates that *GSL6* and *GSL4* are bona fide callose synthases required for SA-dependent and reactive oxygen species (ROS)-dependent plasmodesmata regulation, respectively (Cui and Lee, 2016). BR-enhanced plant immunity was accompanied by increased callose accumulation (Xiong et al., 2020). However, BR enhances plant immunity *via* inducing callose accumulation that lacks direct evidence. To gain more insight into BR-activated plant immunity, we investigated the effects of BR-induced callose accumulation on pathogen resistance. We identified a key component *GLUCAN SYNTHASE-LIKE 8 (GSL8)*, which was a direct target of BES1, and its expression was promoted by BRs/BES1. BR-induced pathogen resistance correlated with callose enrichment, and *GSL8* played key roles in BR-mediated resistance against *Pseudomonas syringae* pv. *tomato* DC3000 (*Pst* DC3000).

## Materials and Methods

### Plant Materials and Growth Conditions

The *Arabidopsis* transgenic and mutant plants *BRI1OX, DWF4OX, det2, bes1-D*, *BES1-RNAi*, and *gsl8-1* are in Col-0 background, and the *DET2OX* is in Wassilewskija (WS) background. *GSL8* T-DNA insertion mutant *gsl8-1* (SALK_111094) was obtained from Arabidopsis Biological Resource Center (ABRC), and the details were described previously (Chen et al., 2009). *Arabidopsis* plants used in the study were sterilized using 70% (v/v) ethanol and 0.1% (v/v) Triton X-100, plated on 1/2 Murashige and Skoog (1/2 MS) medium, vernalized at 4°C for 2 days in the dark, were incubated for 6 h in the light (150 μmol m^–2^ s^–1^) at 22°C for germination, and then grown under a long-day condition (22°C, 16-h light/8-h dark).

### Chemical Treatments and Pathogen Inoculation

The *Arabidopsis* leaves were pretreated by BL or BRZ (1 μM with 0.02% Tween 20) at 12 h before infection. *Pst* DC3000 or *Pst* hrcC was cultured on the solid King’s B (KB) medium (peptone 20 g/L, glycerin 10 ml/L, K_2_HPO_4_ 1.5 g/L, MgSO_4_⋅7H_2_O 1.5 g/L, and rifampicin 50 mg/L) at 28°C for 24–48 h. Bacteria were scraped off the plates and suspended in 10 mM MgCl_2_ to OD600 of 0.02, inoculating 10 mM MgCl_2_ without bacteria as mock (Choi et al., 2010), photographed at 3 and 5 days postinoculation (dpi). Pathogen growth analyzed at 1 dpi, and 0.8 cm^2^ leaf disks were taken by puncher, washed by 15% H_2_O_2_ for 3 min, and then washed with sterile distilled H_2_O. The leaf disks were then continuously diluted by water and plated onto the KB medium.

### Analysis of Chlorophyll Fluorescence

The analysis of chlorophyll fluorescence was described previously (Deng et al., 2015). In brief, chlorophyll fluorescence was determined with an imaging pulse amplitude-modulated fluorometer (IMAG-MINI, Heinz Walz, Germany). For the measurement of *F*v/*F*m, plants were dark-adapted for 30 min. Minimal fluorescence (*F*o) was measured during the weak measuring pulses, and maximal fluorescence (*F*m) was measured by a 0.8-s pulse of light at about 4,000 μmol m^–2^ s^–1^. An actinic light source was then applied to obtain steady-state fluorescence yield (*F*s), after which a second saturation pulse was applied for 0.7 s to obtain light-adapted maximum fluorescence (*F*m′). *F*v*/F*m and non-photochemical quenching (NPQ) were calculated as *F*m−*F*o/*F*m and (*F*m/*F*m′)^–1^, respectively.

### Superoxide Staining and Antioxidant Enzyme Activity Determinations

For superoxide staining, leaves were stained by nitroblue tetrazolium (NBT; 0.5 mg/ml) for 2 h and then soaked in boiling ethanol (95%) until the green color of the leaves faded. The Micro Superoxide Anion Assay Kit (BC1295, Solarbio, Beijing, China) was used to measure the superoxide content. The Catalase (CAT) Activity Assay Kit (BC0205, Solarbio), the Superoxide Dismutase (SOD) Activity Detection Kit (BC0170, Solarbio), the Peroxidase (POD) Activity Detection Kit (BC0090, Solarbio), and the Ascorbate Peroxidase (APX) Activity Assay Kit (BC0220, Solarbio) were used to detect the activity of CAT, SOD, POD, and APX, respectively.

### Callose Deposition Staining

Callose deposition staining was observed at 1 dpi; leaves were cleared by decolorizing solution (acetic acid:ethanol = 1:3) for 12 h, then washed by water, and stained by aniline blue (150 mM K_2_HPO_4_ (pH 9.5) supplemented with 0.01% aniline blue). Callose deposition was observed by fluorescence microscope with DAPI filter (MDG41, Leica). ImageJ software^1^ was used to count the number of callose depositions (Choi et al., 2010).

### RNA Extraction and Quantitative Reverse-Transcription PCR

Total RNA was extracted by the Total RNA Extraction Kit (Solarbio). For quantitative reverse-transcription PCR (qRT-PCR), cDNA was prepared using PrimeScript™ RT Reagent Kit (Takara). Gene expression was performed using the SYBR Green PCR Master Mix (Invitrogen). The CFX Connect Real-Time System (Bio-Rad, Hercules, CA, United States) was used for the qRT-PCR analysis. For each sample, three replicates were performed, and the expression levels were normalized to those of *ACTIN2.* The primers used for qRT-PCR are listed in Supplementary Table 1.

### Chromatin Immunoprecipitation Assays

Chromatin immunoprecipitation (ChIP) assays were performed as previously described (Zou et al., 2019). In brief, 4-week-old Col-0 was cross-linked with formaldehyde, and 125 mM glycine stopped the reaction. Chromatin was sonicated to produce approximately 0.3 kbp DNA fragments. The sonicated protein-DNA complexes were precipitated with an anti-BES1 antibody. After incubation with protein A beads, the beads were further washed with low salt and high salt buffer and reverse cross-linked with 200 mM NaCl. After removing proteins with proteinase K, DNA fragments were purified by phenol-chloroform extraction and ethanol precipitation. The DNA fragments were dissolved in TE buffer (10 mM Tris–HCl pH 8.0, 1 mM EDTA) and used as qPCR templates for the real-time system. TA3 fragment served as a normalization for the qPCR analysis.

### Transient Transcription Assay

*Arabidopsis* mesophyll cell protoplasts were prepared and transformed as described previously (Li et al., 2020). For luciferase (LUC) assays, we cloned the promoters of *GSL8* into the pGreen II 0800 vector and the full-length coding sequence (CDS) of *BES1* into the pCAMBIA1307. The constructs used in this study were mentioned in the previous studies (Li et al., 2020; Zhang et al., 2021). Plasmids were singly or co-transformed into *Arabidopsis* protoplasts. LUC activities were measured using a Luciferase Assay System (Promega) after 16 h, and the data were normalized to *REN* activity. The experiments were repeated three times with similar results.

### Accession Numbers

Sequence data from this study can be found in the Arabidopsis Genome Initiative database under the following accession numbers: *GSL8* (AT2G36850), *BES1* (AT1G19350), *BRI1* (AT4G39400), *DWF4* (AT3G50660), and *DET2* (AT2G38050).

### Statistical Analysis

The experimental data were statistically analyzed using three or more averages, using one-way ANOVA, and considered significant when *P* < 0.05.

## Results

### Brassinosteroids Enhance the Resistance of *Arabidopsis* Against *Pseudomonas syringae* pv. *tomato* DC3000

**FIGURE 1:**
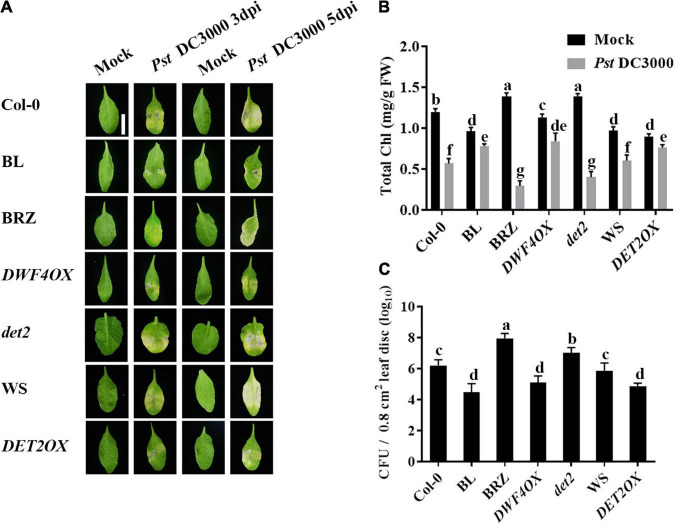
Brassinosteroids (BRs) increase resistance to *Pst* DC3000. **(A)** Typical *Pst* DC3000 infection symptoms in Col-0, BL-treated, BRZ-treated, *DWFOX*, *DET2OX*, WS, and *det2* plants. Pictures were taken at 3 and 5 dpi, respectively. Bar, 1.00 cm. **(B)** Total chlorophyll content in inoculated leaves was detected in planta at 3 dpi. **(C)** Bacterial growth in the inoculated leaves was detected in planta. Bacteria were isolated from plants at 1 dpi and quantified with gradient dilution assays. Bars represent mean ± SD obtained from three biological replicates per genotype and time point, chlorophyll content or bacterial growth measured from five leaves of each genotype and treatment were pooled for one replicate. Significant differences (*P* < 0.05) are denoted by different lowercase letters.

To study the roles of BRs in plant–pathogen interactions, we examined the effect of brassinolide (BL; the most active BRs) or brassinazole (BRZ, a specific BR biosynthesis inhibitor) treatments on *Pst* DC3000 resistance in *Arabidopsis*. At 3 and 5 dpi, plants presented typical disease symptoms and chlorotic leaves (Ishiga et al., 2009). It spread more rapidly in treatment with 1 μM BRZ, while the application of 1 μM BL significantly enhanced *Pst* DC3000 resistance (Figures 1A,B). Then, we detected the pathogen accumulation in infected leaves at 1 dpi, and BL-treated plants showed less bacterial counts than mock and BRZ-treated plants (Figure 1C). Then, we used various BR biosynthesis genes and transgenic and mutant plants, including BR biosynthesis gene *DWF4* overexpression transgenic line (*DWF4OX*), BR biosynthesis gene *DET2* knock out mutant (*det2*), and *DET2* overexpression transgenic line (*DET2OX*), to investigate the role of BR biosynthesis in plant immunity. BR biosynthesis-enhanced transgenic plants *DWF4OX* and *DET2OX* showed higher resistance to *Pst* DC3000 infection, while BR biosynthesis-deficient mutant *det2* showed susceptibility to infection (Figure 1). It demonstrated that BRs enhance plant resistance to *Pst* DC3000.

### Brassinosteroids Alleviate Photosystem Damage After *Pseudomonas syringae* pv. *tomato* DC3000 Infection

**FIGURE 2:**
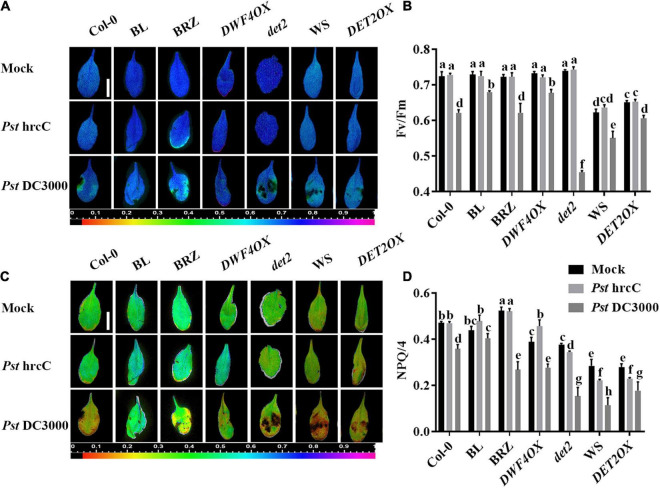
Brassinosteroids alleviate photosystem damage after *Pst* DC3000 infection. Images of the maximum photosystem II (PSII) quantum yield (*F*v/*F*m) **(A)** and non-photochemical quenching (NPQ)/4 **(C)** in the leaves infected by *Pst* DC3000 or *Pst* hrcC at 3 dpi. Bar, 1.00 cm. Average values of *F*v/*F*m **(B)** and NPQ/4 **(D)** for the respective chlorophyll fluorescence images. *F*v/*F*m or NPQ/4 measured from 8 to 10 leaves and three biological repeats. Significant differences (*P* < 0.05) are denoted by different lowercase letters.

Two typical indicators of photosystem II (PSII) photochemistry activity, namely, *F*v/*F*m (the maximal quantum efficiency of PSII) and NPQ, were detected to test the degree of damage to the light system caused by bacterial inoculation. As shown in Figure 2, there were no significant differences in *F*v/*F*m (Figures 2A,B) and NPQ (Figures 2C,D) in unchallenged or *Pst* hrcC inoculated plants. On *Pst* DC3000 infection, compared with mock-treated wild-type plants, both *F*v/*F*m (Figures 2A,B) and NPQ (Figures 2C,D) decreased in all of the plants, but BRZ-treated and *det2* plants decreased more. It indicated that BRs played a critical role in protecting plant photosystem against *Pst* DC3000 infection.

### Involvement of Antioxidant System in Brassinosteroids-Induced *Pseudomonas syringae* pv. *tomato* DC3000 Defense

**FIGURE 3:**
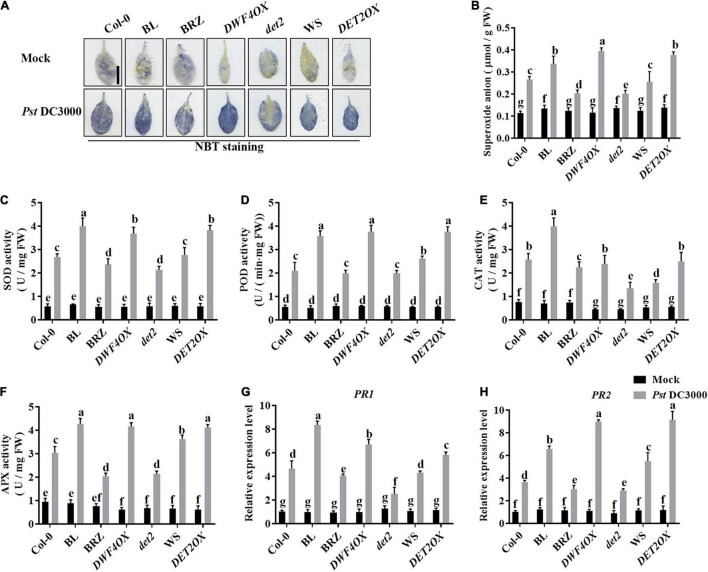
Antioxidant system induced by BRs after infection. **(A)** Nitroblue tetrazolium (NBT) staining for observing superoxide accumulation at 1 dpi. **(B)** The superoxide content in the infected leaves at 1 dpi. Bar, 1.00 cm. The activities of the antioxidant enzymes superoxide dismutase (SOD) **(C)**, peroxidase (POD) **(D)**, catalase (CAT) **(E)**, and ascorbate peroxidase (APX) **(F)**. Bars represent mean ± SD obtained from three biological replicates per genotype and time point, superoxide content or the activities of the antioxidant enzymes measured from five leaves of each genotype and treatment were pooled for one replicate. **(G,H)** Relative expression levels of defense-related genes *PR1* and *PR2.* The expression of *ACTIN2* was used as an internal reference. Data presented are mean ± SD from three independent experiments. Significant differences (*P* < 0.05) are denoted by different lowercase letters.

Pathogen infection promotes the accumulation of ROS in plants (Deng et al., 2015). Then, we explored the effects of BRs on antioxidant systems when plants were incubated with *Pst* DC3000. We detected the accumulation of superoxide by NBT staining (Figure 3A) and quantified it by biochemical testing (Figure 3B). The accumulation of superoxide had no significant difference in unchallenged plants but increased after *Pst* DC3000 infection. The accumulation of superoxide was higher in BL-treated, *DWF4OX*, and *DET2OX* plants but lower in BRZ-treated and *det2* plants. The enzyme activity of several antioxidative enzymes, such as SOD, POD, CAT, and APX, and the relative expression levels of defense-related genes (*PR1* and *PR2*) were also detected. *Pst* DC3000 infection increased the activities of all these antioxidative enzymes (Figures 3C–F) and relative expression levels of defense-related genes (Figures 3G,H), and the increase was higher in BL-treated and BR biosynthesis-enhanced transgenic plants and lower in BRZ-treated and BR biosynthesis-deficient mutant plants. All these results illustrated that BRs-induced defense of *Pst* DC3000 was related to the antioxidant system.

### Brassinosteroids Enhance Callose Deposition After *Pseudomonas syringae* pv. *tomato* DC3000 Infection

**FIGURE 4:**
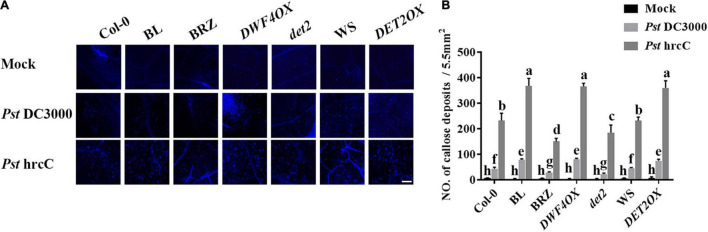
Brassinosteroids induce callose deposition after infection. **(A)** Callose deposition in infected leaves at 1 dpi. Callose deposition was visualized by fluorescence microscopy. Bar, 200 μm. **(B)** Number of callose deposition in the 5.5 mm^2^ microscopic fields. Callose deposition was counted in 8–12 microscopic fields of 5.5 mm^2^ from 8 to 12 different leaves and three biological repeats. Significant differences (*P* < 0.05) are denoted by different lowercase letters.

The induction of callose deposition indicates the activation of basal defenses. As *Pst* DC3000 suppressed callose deposition, to understand the nature of resistance induced by BL, callose deposition was observed at the *Pst* hrcC infection leaves. When infected with *Pst* hrcC, compared with wild-type plants, BRZ-treated and BR biosynthesis-deficient mutant showed significantly lower levels of callose deposition, while BL-treated and BR biosynthesis-enhanced transgenic plants accumulated a higher number of callose deposition in leaves (Figures 4A,B). It indicated that BRs induced callose accumulation against *Pst* DC3000 infection.

### Brassinosteroid Signaling Positively Regulates Disease Resistance to *Pseudomonas syringae* pv. *tomato* DC3000

**FIGURE 5:**
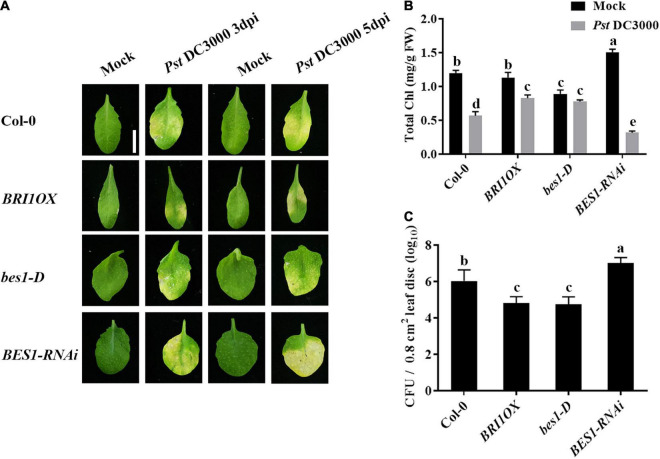
Brassinosteroid signaling positively regulates plant defense. **(A)** Typical *Pst* DC3000 infection symptoms in Col-0, *BRI1OX*, *bes1-D*, and *BES1-RNAi*. Pictures were taken at 3 and 5 dpi, respectively. Bar, 1.00 cm. **(B)** Total chlorophyll content in inoculated leaves was detected in planta at 3 dpi. **(C)** Bacterial growth in the inoculated leaves was detected in planta. Bacteria were isolated from plants at 1 dpi and quantified with gradient dilution assays. Bars represent mean ± SD obtained from three biological replicates per genotype and time point, chlorophyll content or bacterial growth measured from five leaves of each genotype and treatment were pooled for one replicate. Significant differences (*P* < 0.05) are denoted by different lowercase letters.

**FIGURE 6:**
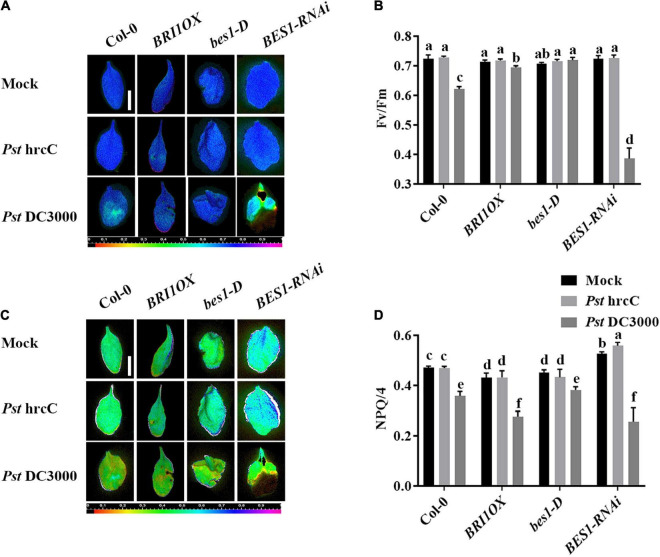
Brassinosteroid signaling enhances plant defense by alleviating photosystem damage. Images of the maximum PSII quantum yield (*F*v/*F*m) **(A)** and NPQ/4 **(C)** in the leaves infected by *Pst* DC3000 or *Pst* hrcC at 3 dpi. Bar, 1.00 cm. Average values of *F*v/*F*m **(B)** and NPQ/4 **(D)** for the respective chlorophyll fluorescence images. *F*v/*F*m or NPQ/4 measured from 8 to 10 leaves and three biological repeats. Significant differences (*P* < 0.05) are denoted by different lowercase letters.

**FIGURE 7:**
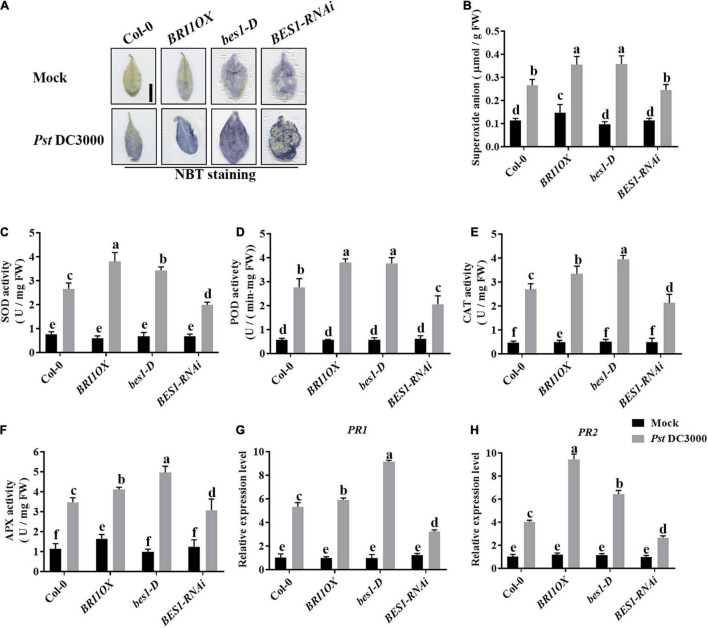
Brassinosteroid signaling enhances the antioxidant system to defend against pathogens. **(A)** NBT staining for observing superoxide accumulation at 1 dpi in Col-0, *BRI1OX*, *bes1-D*, and *BES1-RNAi.* Bar, 1.00 cm. **(B)** The superoxide content in the infected leaves at 1 dpi. The activities of the antioxidant enzymes SOD **(C)**, POD **(D)**, CAT **(E)**, and APX **(F)**. Bars represent mean ± SD obtained from three biological replicates per genotype and time point, superoxide content or the activities of the antioxidant enzymes measured from five leaves of each genotype and treatment were pooled for one replicate. **(G,H)** Relative expression levels of defense-related genes *PR1* and *PR2.* The expression of *ACTIN2* was used as an internal reference. Data presented are mean ± SD from three independent experiments. Significant differences (*P* < 0.05) are denoted by different lowercase letters.

**FIGURE 8:**
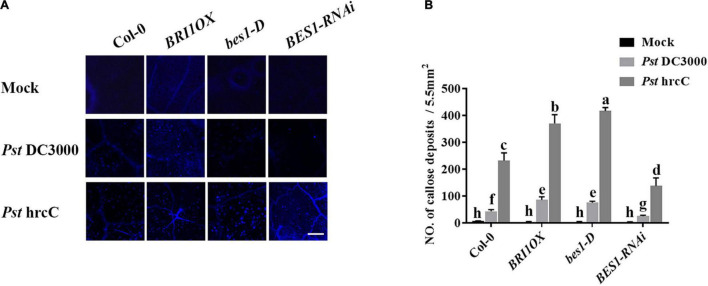
Brassinosteroid signaling induces callose deposition after infection. **(A)** Callose deposition in Col-0, *BRI1OX*, *bes1-D*, and *BES1-RNAi* infected leaves at 1 dpi. Callose deposition was visualized by fluorescence microscopy. Bar, 200 μm. **(B)** Number of callose deposition in the 5.5 mm^2^ microscopic fields. Callose deposition was counted in 8–12 microscopic fields of 5.5 mm^2^ from 8 to 12 different leaves and three biological repeats. Significant differences (*P* < 0.05) are denoted by different lowercase letters.

To further investigate the roles of BRs in plant disease resistance, we analyzed the effects of different BR signaling components and transgenic and mutant plants in *Pst* DC3000 resistance. BR receptor BRI1 overexpression transgenic line (*BRI1OX*), BES1 gain-of-function mutant (*bes1-D*), and BES1 RNA interference transgenic line (*BES1-RNAi*) were used in the future study. *BRI1OX* and *bes1-D* displayed increased *Pst* DC3000 resistance, including fewer disease symptoms (Figures 5A,B), less bacterial accumulation (Figure 5C), alleviated photosystem damage (Figures 6A–D), enhanced antioxidant system (Figures 7A–F), and increased defense-related gene expression (Figures 7G,H), while *BES1-RNAi* displayed the opposite. Then, we analyzed callose deposition in different transgenic and mutant plants after infection. As shown in Figures 8A,B, after infecting with *Pst* hrcC, *BES1-RNAi* showed significantly lower levels of callose deposition, while more callose accumulated in the leaves of *BRI1OX* and *bes1-D*. These results indicated that BR signaling positively regulated plant resistance to *Pst* DC3000.

### GLUCAN SYNTHASE-LIKE 8 Is a Direct Target of BRI1-EMS-SUPPRESSOR 1

**FIGURE 9:**
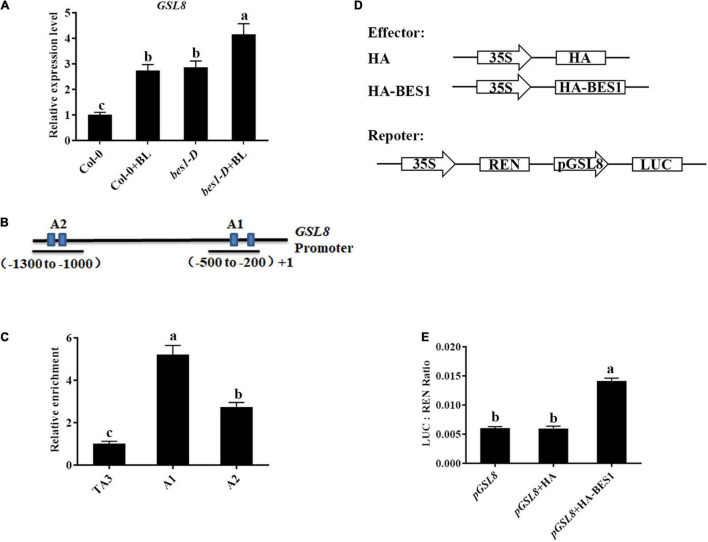
*GLUCAN SYNTHASE-LIKE 8* is a direct target of BES1. **(A)** Detection of *GSL8* expression in Col-0 or *bes1-D* with/without BL treatment. Quantitative reverse-transcription PCR (qRT-PCR) was performed using 4-week-old plants treated with or without 1 μM BL. Data presented are mean ± SD from three independent experiments. **(B)** Schematic representation of *GSL8* promoter. **(C)** Chromatin immunoprecipitation (ChIP)-quantitative PCR (qPCR) assay using 4-week-old Col-0 seedlings. TA3 was used as an internal control. Data presented are mean ± SD from three independent experiments. **(D)** Schematic diagrams of the reporters and effectors used in the transient transactivation assays. **(E)** Transient dual-luciferase (LUC) reporter assays show that BES1 enhances the promoter activity of *GSL8*. The relative LUC activities were calculated by normalizing the LUC values against Renilla LUC (REN). Data presented are mean ± SD from three independent experiments. Significant differences (*P* < 0.05) are denoted by different lowercase letters.

Previous ChIP-chip studies have shown that *GSL8* was a direct target of BES1 and induced by BRs (Yu et al., 2011) (*Arabidopsis* eFP Browser^2^), and *GSL8*-deficient mutants had short hypocotyls (Chen et al., 2009) which are typical BR-deficient phenotypes. Thus, we hypothesized that *GSL8* was a direct target of BES1 in BR-induced plant immunity. To confirm the result, qRT-PCR experiments were performed. The expression of *GSL8* was increased in Col-0 seedlings after BL treatment. In addition, the expression of *GSL8* increased to 294% without exogenous BL in *bes1-D* and even more increased with BL treatment (Figure 9A). Then, the ChIP experiments were performed using an anti-BES1 antibody to confirm whether *GSL8* is a direct target of BES1. TA3, a retrotransposable element, was used as the internal control. In the promoter of *GSL8*, there are two putative BES1 binding fragments at the promoter of *GSL8* (Figure 9B). Results of ChIP-qPCR showed that BES1 was enriched significantly at the A1 and A2 regions of *GSL8* which contain a typical E-box (CANNTG). We then expressed *GSL8* promoter:LUC reporter gene in tobacco leaves. When co-expressed with BES1, *GSL8* pro:LUC gene expression was induced (Figures 9D,E). Taken together, our results demonstrated that *GSL8* was a direct target of BES1, and its expression was induced by BES1.

### Brassinosteroid-Induced Disease Resistance Partially Depends on *GLUCAN SYNTHASE-LIKE 8*

**FIGURE 10:**
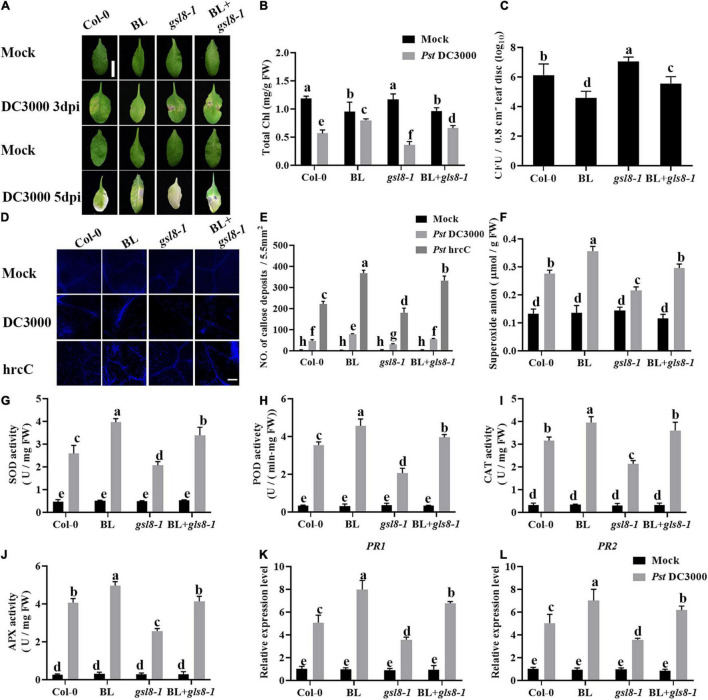
Brassinosteroid-induced disease resistance partially depends on *GSL8*. **(A)** Typical *Pst* DC3000 infection symptoms in Col-0, BL, *gsl8-1*, and BL + *gsl8-1.* Pictures were taken at 3 and 5 dpi, respectively. Bar, 1.00 cm. **(B)** Total chlorophyll content in inoculated leaves was detected in planta at 3 dpi. **(C)** Bacterial growth in the inoculated leaves was detected in planta. Bacteria were isolated from plants at 1 dpi and quantified with gradient dilution assays. **(D)** Callose deposition in infected leaves at 1 dpi. Callose deposition was visualized by fluorescence microscopy. Bar, 200 μm. **(E)** Number of callose deposition in the 5.5 mm^2^ microscopic fields. Callose deposition was counted in 8–12 microscopic fields of 5.5 mm^2^ from 8 to 12 different leaves and three biological repeats. **(F)** The superoxide content in the infected leaves at 1 dpi. The activities of the antioxidant enzymes SOD **(G)**, POD **(H)**, CAT **(I)**, and APX **(J)**. **(K,L)** Relative expression levels of defense-related genes *PR1* and *PR2.* Data presented are mean ± SD from three independent experiments. The expression of *ACTIN2* was used as an internal reference. Significant differences (*P* < 0.05) are denoted by different lowercase letters.

To further investigate the connection of BR-induced disease resistance and *GSL8*, a *GSL8* T-DNA insertion mutant *gsl8-1* was used for the follow-up experiments. We analyzed the effects of BL on *Pst* DC3000 resistance in Col-0, BL, *gsl8-1*, and BL + *gsl8-1*. As shown in Figures 10A,B, *gsl8-1* mutant showed more obvious disease symptoms, after being treated with BL, the symptoms were relieved but still severer than BL-treated wild-type plants. Then, we detected bacterial growth in infected leaves. As the same as symptoms, bacterial counts in *gsl8-1* were higher than wild-type. After being treated with BL, bacterial counts in *gsl8-1* were obviously relieved but still higher than BL-treated wild-type plants (Figure 10C). We found BR-induced callose accumulation against *Pst* DC3000, and *GSL8* is one of the callose synthases, whether the susceptibility of *gsl8-1* is due to callose synthesis blocked? Thus, we detected the callose accumulation in Col-0 and *gsl8-1* with/without BL treatment after being infected with *Pst* hrcC. Compared with Col-0, callose accumulation in *gsl8-1* was decreased 38%, although increased 28% in BL + *gsl8-1*, and increased 58% in BL-treated wild-type plants (Figures 10D,E). Then, we detected the superoxide contents, antioxidative enzyme activities, and defense-related gene expressions in Col-0 and *gsl8-1* with/without BL treatment after infection. Compared with Col-0, superoxide contents in *gsl8-1* were significantly decreased to 19% (Figure 10F). Also, the antioxidative enzyme activities (Figures 10G–J) and defense-related gene expressions (Figures 10K,L) showed similar trends. These results suggested that BR-induced disease resistance partially depends on *GSL8*.

## Discussion

Plant immunity is regulated by a powerful and efficient phytohormone regulatory network (Pieterse et al., 2009), ET, JA, SA, ABA, CK, auxin, and BRs have been reported to positively or negatively regulate plant immunity (Pieterse et al., 2014). Previous studies have demonstrated that BAK1 leads to the initiation of innate immunity (Chinchilla et al., 2007; Heese et al., 2007), and BSK1 promotes disease resistance by phosphorylating a site in the N terminus of MAPK5 (Yan et al., 2018; Zhou and Zhang, 2020). Meanwhile, as a direct substrate of MEK6, BES1 plays a critical role in plant immunity (Kang et al., 2015). However, systematic research about the molecular mechanism of BRs in regulating plant immunity from BR biosynthesis to signaling perception and response is poor, and the downstream potential component still needs to be identified. In this study, we used various BR biosynthesis and signaling transgenic and mutant plants to study the mechanism of how BRs work in plant immunity. BL-treated and BR biosynthesis-enhanced transgenic plants *DWF4OX* and *DET2OX* displayed significantly enhanced plant resistance to *Pst* DC3000, and *BRI1* overexpression transgenic plant *BRI1OX* and BES1 gain of function mutant *bes1-D* increased *Pst* DC3000 resistance, while BRZ-treated, *det2*, and *BES1-RNAi* showed reduced resistance (Figures 1, 5). All the data indicate that BRs are comprehensively involved in plant immunity comprehensively, from biosynthesis to signaling perception and response.

Biotic and abiotic stress often accompanies the production of ROS, which plays a critical role in stress responses. Recent studies indicate that BR-induced ROS accumulation enhances plant tolerance to abiotic stress, and BRs enhance virus resistance through MEK2-salicylic acid-induced protein kinase (SIPK) cascade and respiratory burstoxidase homolog B (RBOHB)-dependent ROS burst (Deng et al., 2015, 2016). In this study, we found that *BRI1OX*, *DWF4OX*, *DET2OX*, and *bes1-D* performed higher ROS accumulation after *Pst* DC3000 inoculation (Figures 3, 7), suggesting BRs induced *Pst* DC3000 resistance partially by stimulating the production of ROS.

As a core transcription factor in BR signaling, BES1 regulates plant growth and development by influencing BR-regulated gene expression (Nolan et al., 2020). Previous studies have revealed that BR antagonizes JA responses (He et al., 2020; Liao et al., 2020; Song et al., 2021). BES1 suppresses JA-induced transcription of *PDF1.2s* and indole-GS biosynthesis genes during pathogen infection and herbivore feeding (Liao et al., 2020), while *Pst* bacteria are able to synthesize the JA mimic coronatine, and our results reinforce the concept that BR antagonizes the JA responses. Callose is involved in various plant developmental processes and stress responses, and its biosynthesis is regulated by the family of *GSL* genes (Ellinger and Voigt, 2014), and several *GSL* genes are induced during plant immunity. BR-induced plant resistance to *Pst* DC3000 accompanied with callose accumulation (Figures 4, 8), whether BR directly regulates callose synthesis to enhance plant defense remains unknown. Our research found that *GSL8* was a direct target of BES1 and its expression was induced by BRs/BES1 (Figure 9), *GSL8*-deficient mutant *gsl8-1* showed a susceptible phenotype, and BR-induced callose accumulation in *gsl8-1* was blocked (Figures 10A–E), indicating that *GSL8*-induced callose accumulation was important to BR-induced plant defense. Plants treated with BL rescued susceptible phenotype and callose deposition deficient in *gsl8-1* but still lower than BL-treated wild-type plants (Figures 10A–E), suggesting that there may be other components take part in BR-induced plant defense. These results suggest that BR-induced pathogen resistance partly depends on the *GSL8*-mediated callose accumulation.

## Conclusion

In summary, our research demonstrated a plant defense pathway mediated by BR signaling, and BR signaling is involved in plant immunity comprehensive, from biosynthesis to signaling perception and response. The core transcription factor BES1 positively regulates pathogen-induced callose accumulation *via* a glucan synthase gene *GSL8*. BR-induced pathogen resistance partly depends on the BR-induced BES1-GSL8 cascade to mediate callose accumulation.

## Data Availability Statement

The original contributions presented in the study are included in the article/Supplementary Material, further inquiries can be directed to the corresponding author.

## Author Contributions

JX and FY designed the research and wrote the manuscript. JX and XW performed most of the experiments with the assistance of LC. XX and MR contributed to the analytical tools. FY analyzed the data. MR and XX undertook most of the manuscript revisions. All authors contributed to the article and approved the submitted version.

## Conflict of Interest

LC is employed by the Sichuan Huitai Agriculture Technology Co. Ltd. The remaining authors declare that the research was conducted in the absence of any commercial or financial relationships that could be construed as a potential conflict of interest.

## Publisher’s Note

All claims expressed in this article are solely those of the authors and do not necessarily represent those of their affiliated organizations, or those of the publisher, the editors and the reviewers. Any product that may be evaluated in this article, or claim that may be made by its manufacturer, is not guaranteed or endorsed by the publisher.

## Footnotes

1
https://imagej.nih.gov/ij/download.html

2
http://bbc.botany.utoronto.ca/efp/cgi-bin/efpWeb.cgi

## Abbreviations

BRs: brassinosteroids
BL: brassinolide
BRZ: brassinazole
*Pst*: *Pseudomonas syringae* pv. *tomato*
*GSL8*: *GLUCAN SYNTHASE-LIKE 8*
*BRI1*: *BR INSENSITIVE 1*
*BES1*: *BRI1-EMS-SUPPRESSOR 1*
*DWF4*: *DWARF 4*
*DET2*: *DEETIOLATED 2*
PSII: photosystem II
NPQ: non-photochemical quenching
ROS: reactive oxygen species
NBT: nitroblue tetrazolium
SOD: superoxide dismutase
POD: peroxidase
CAT: catalase
APX: ascorbate peroxidase
qRT-PCR: quantitative reverse-transcription PCR
ChIP: chromatin immunoprecipitation.
