# Supplementary material for: Brassinosteroids Positively Regulate Plant Immunity via BRI1-EMS-SUPPRESSOR 1-Mediated GLUCAN SYNTHASE-LIKE 8 Transcription
Source: Front Plant Sci. 2022 Mar 24;13:854899. doi: 10.3389/fpls.2022.854899 (PMC8988940; doi:10.3389/fpls.2022.854899)
Supplement: Supplementary file 1 [file Table_1.DOCX]

Supplementary Material

Supplementary Table S1**.** Primer sequences used in this study.

| **Gene** | **F primer (5’→3’)** | **R primer (5’→3’)** |  |
| --- | --- | --- | --- |
| *PR1* | CGAGAAGGCTAACTACAACTACG | ACACCTCACTTTGGCACATC | For qPCR |
| *PR2* | CCTTGCTCGTGAATCTCTACC | GTGAACAGAGCGTAGTCTAGATG |  |
| *GSL8* | TCATATTTTCTCCGATCGCCC | CTTCTACTTCCACACTAACTTCTCT |  |
| *GSL8 pro* | TATCAATATCTTGTCAATTTTAATT | AATTTTCAAATCCTAGAGGAGGA | For luciferase assays |
| *BES1* | ATGAAAAGATTCTTCTATAAT | TCAACTATGAGCTTTACCATT |  |
| *TA3* | GATTCTTACTGTAAAGAACATGGCATTGAGAGA | TCCAAATTTCCTGAGGTGCTTGTAACC | For ChIP-qPCR |
| *GSL8 pro* A1 | ACAATTTTATCTGAAACTAGAGATA | ACTACTACAGTCTTCCGCTCT |  |
| *GSL8 pro* A2 | CTTTTGAGAGACGTAAAGTTTGA | TAAATTTACCATAAATATGATGTAC |  |
